# Improvement of Phase Change Materials (PCM) Used for Solar Process Heat Applications

**DOI:** 10.3390/molecules26051260

**Published:** 2021-02-26

**Authors:** Cristina Prieto, Anton Lopez-Roman, Noelia Martínez, Josep M. Morera, Luisa F. Cabeza

**Affiliations:** 1 Abengoa Energía, c/Energía Solar 1, 41012 Sevilla, Spain; anton.lopez@abengoa.com (A.L.-R.); noelia.martinez@abengoa.com (N.M.); 2 Department of Energy Engineering, University of Seville, Camino de los Descubrimientos s/n, 41092 Sevilla, Spain; 3 GREiA Research Group, Universitat de Lleida, Pere de Cabrera s/n, 25001 Lleida, Spain; josepmaria.morera@udl.cat

**Keywords:** solar process heat, thermal energy storage, phase change material, metal wool, effective thermal conductivity enhancement, inert atmosphere

## Abstract

The high intermittency of solar energy is still a challenge yet to be overcome. The use of thermal storage has proven to be a good option, with phase change materials (PCM) as very promising candidates. Nevertheless, PCM compounds have typically poor thermal conductivity, reducing their attractiveness for commercial uses. This paper demonstrates the viability of increasing the PCM effective thermal conductivity to industrial required values (around 4 W/m·K) by using metal wool infiltrated into the resin under vacuum conditions. To achieve this result, the authors used an inert resin, decoupling the specific PCM material selection from the enhancement effect of the metal wools. To ensure proper behavior of the metal wool under standard industrial environments at a broad range of temperatures, a set of analyses were performed at high temperatures and an inert atmosphere, presenting a thorough analysis of the obtained results.

## 1. Introduction

Several industrial sectors, such as the food industry or the chemical industry, are good candidates for the integration of solar energy as their main energy source for process heat generation [1]. Nevertheless, the characteristic discontinuity of solar energy requires the use of storage systems to ensure the availability of thermal energy, making these thermal energy storage (TES) systems a necessity for the correct implementation of solar energy in the industry. The use of phase change materials (PCM) to store solar energy in different applications was developed by many researchers in the last two decades, and the use of this technology in the so-called high-temperature applications is increasing [REF]. Within this context, high-temperature applications are those using storage at temperatures higher than 150 °C, going up to 1000 °C in applications such as concentrated solar power, and being between 200 °C and 400 °C in applications that use solar energy in industry or for industrial waste heat recovery [2,3,4,5,6]. Among the available TES technologies, latent TES is seen as a good potential candidate due to its ability to provide heat at a constant temperature, matching the requirement of steam generation, one of the most used heat transfer fluids (HTF) in the industry [7]. Nevertheless, most authors agree that one of the main drawbacks of the use of PCM when high power is needed is the low thermal conductivity of the materials [8,9]. The use of graphite foams with high porosity is the highly used options in the literature. Here, the main challenge is the infiltration of the PCM in the graphite matrix. When highly conductive particles are added to the PCM, the main drawback is the deposition of the particles after several thermal cycles.

Due to this low thermal conductivity, there is an increasing interest to develop new PCM compounds that have higher effective thermal conductivity than pure PCM. There are several options to do so [3], such as fins or heat pipes, aiming to increase exchange surfaces or the combination of highly conductive materials within the PCM (graphite, metal foams, or nano-additives). Of those, fins are commonly used in several industries with successful results, but for the PCM case, they present a challenge when considering transient behaviors, coupled with the difficult selection of fin shape and positioning [8]. When analyzing the integration of conductive materials into the PCM, graphite foams are the solution with higher acceptance in academia [10] due to the high temperatures required for process heat. The main drawback of this compound is the possibility of PCM leakage [11], not to mention the difficulties of including the PCM into the graphite matrix. Organic PCMs (such as paraffin) can be incorporated via infiltration, with or without vacuum, but inorganic materials need more intricate processes, as described by Cabeza et al. [12]. Finally, graphite composites are expensive, greatly affecting the total PCM cost and reducing its potential industrial use.

Metal wool, on the other hand, is a cheap material with high thermal conductivity that can be used to enhance the conductivity of PCMs. It has already been tested at low-temperature applications, with good potential [13,14]. Recently, Yousef et al. [15,16] studied the use of these wool fibers as a heat transfer enhancement method in a solar still latent heat storage system, and compared its performance with other heat transfer enhancement methods, such as fins. The experimental results showed a 25% productivity increase using metal wool fibers. That is one of the few studies around the capability of metal wools as a heat transfer enhancement method, which has not gathered much attention by academia.

Prieto et al. [17] showed that the use of metal wool is a good low-cost alternative to other thermal enhancement techniques. That study developed a model that validated experimentally the increase in the effective conductivity of the composite formed by salts and metal wool. That study showed the experimental prototype that was developed for the validation of the effective conductivity of the composite formed by NaNO_3_ salts and metal wool. The metal wool used was produced and arranged to ensure the right porosity and packaging to increase the effective thermal conductivity of the mixture by 300%. The model validated confirmed the movement of the fluid during the melting, standardizing the temperature of the molten material and increasing the transference. Prieto et al. [17] also identified the predominant transfer mechanisms: Conductivity and convection, and verified at the same time how the temperature of the material was homogenized by the movements of the fluid during the fusion. The model validated the new composite, with wool and NaNO_3_ as PCM, as one of the most promising materials to be used in applications that need heat to be stored at around 280–300 °C. Additionally, this storage concept can be used with different composite mixtures of wool and inorganic salt to cover the full range of solar energy and waste heat in the industry (200 to 500 °C). For this standardization purpose, it is important to validate which is the correct distribution of the wool that guarantees the increase in conductivity of the composite in the desired direction. Therefore, this study shows a new concept on how to use metal wool to produce a composite, with the analysis carried out to evaluate the concept. This paper tries to characterize the effective conductivity (both axial and radial) of the composite PCM-metal wool in a more relevant scale. It is at this higher scale (TRL 5) where the configuration of the composite needs to be validated. This work analyzes the effective thermal conductivity of the composite PCM-metal wools (90% volume) using two dispositions: (i) A casting method and (ii) an infiltrating technique [18,19]. Both techniques use a specific distribution of PCM-wools able to optimize the axial conductivity of the mix. This study uses an inert resin (Epoxi Resoltech 1050) to facilitate the handling during the tests since it is not focused on a specific mixture but the thermal behavior of the composite. The final selection of the PCM will depend on the final application and the chosen operation temperature of the storage.

Once the constitution process of the composite and the characterization of its effective thermal conductivity improvement are explained, this study analyses the effect of thermal cycling the metal wool in controlled atmospheres to reproduce and validate the behavior of the composite in commercial plants. In this paper, the metal wools are tested for a broad range of temperatures using the typical configuration of an inert atmosphere present in high-temperature PCMs, which is needed due to their corrosive nature at those temperatures [20]. For future applications, once the specific PCM solution is selected, a corrosion analysis of the mixture needs to be performed.

## 2. Results

### 2.1. Composite Production

**Figure 1:**
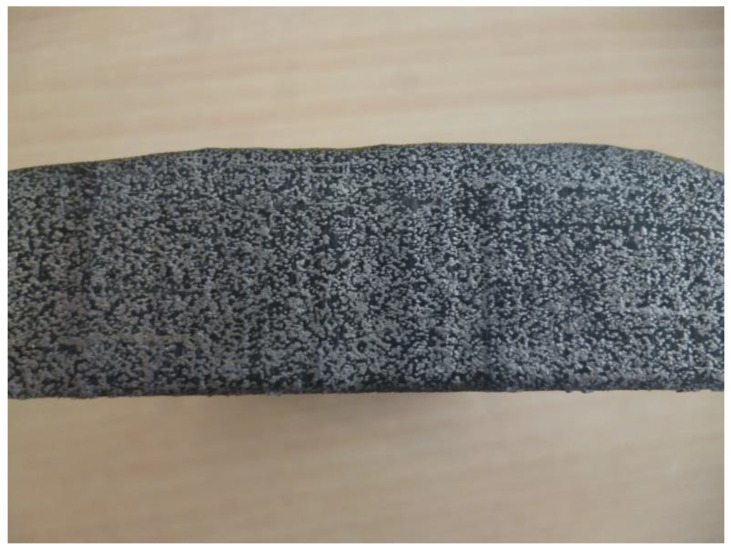
Epoxi resin-metal wool composite produced via vacuum infiltration.

Two processes for the constitution of the mixture PCM-metal wool at 90% volume were evaluated: Casting and vacuum infiltration. The production of the composite via casting did not achieve the targeted proportion of storage material, deeming it unsuccessful, while the production of the metal foam with vacuum infiltration did achieve the expected results (Figure 1).

### 2.2. Effective Thermal Conductivity Evaluation

This study has validated the increase of the effective thermal conductivity of the composite, being it more relevant on the axial direction of the metal wool fiber than on the radial direction, as it could be expected. For this study, the material was considered anisotropic.

With the measured values of the Cp, the thermal conductivity is defined. For each section and using two samples, five values for the thermal conductivity are obtained, both for the axial thermal conductivity and for the radial thermal conductivity.

Thermal conduction in the tested epoxy wool matrix composite material has been found to be anisotropic. Most noticeably, the thermal conductivity of the epoxy wool matrix composite material is very different, by five times, between the direction parallel to the axial layers and the direction perpendicular to the wool layers, as shown in Table 1 and Table 2.The values obtained during the experimental campaign have a mean value of axial effectiveness thermal conductivity of 4.34 W/m·K, and radial effectiveness of 0.77 W/m·K. This measured axial effective thermal conductivity value is very similar to the one calculated via simulations in previous studies [17] and similar to other advanced composites used in other applications [21].

### 2.3. Behavior of the Metal Wool under an Inert Atmosphere

**Figure 2:**
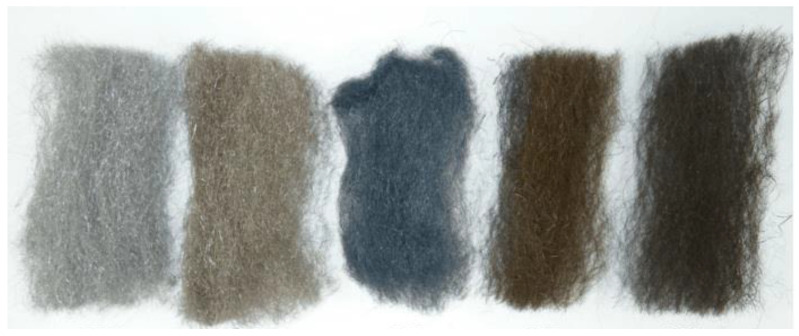
Wool samples after thermal treatment under an inert atmosphere. From left to right: Initial sample, sample treated at 200 °C, sample treated at 300 °C, sample treated at 400 °C, and sample treated at 500 °C.

A visual inspection of the metal wool after the thermal test only shows a variation of the color of the samples, as shown in Figure 2. This color variation hints towards the generation of a superficial oxide layer, which leads to a more thorough analysis of the samples.

**Figure 3:**
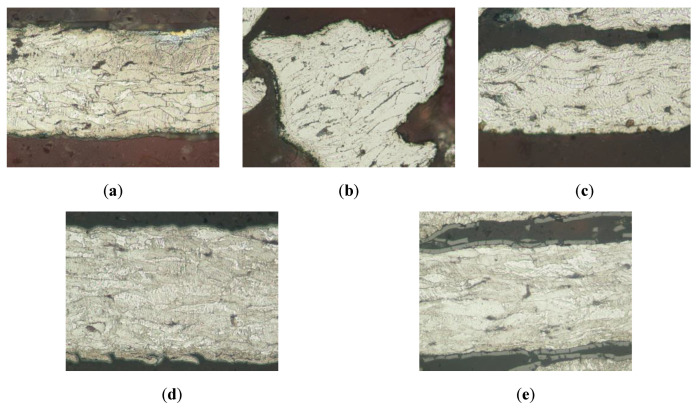
Optical microscopy analysis of the wool samples after thermal treatment under an inert atmosphere. (**a**) Initial sample, (**b**) sample treated at 200 °C, (**c**) sample treated at 300 °C, (**d**) sample treated at 400 °C, and (**e**) sample treated at 500 °C.

The first step to determine the changes in the structure of the samples is to perform an optical microstructure analysis of the thermally treated samples, which is depicted in Figure 3. This analysis shows that all samples have a ferrite base, with an indication of high mechanical deformation due to the anisotropy in the grains and the small perlite colonies. This configuration is characteristic of the fiber generation method, and the absence of any modification after the thermal tests, as shown in Figure 3, indicates material thermal stability. This result was expected since the thermal treatment is performed at temperatures below the recrystallization temperature for steel, which happens above 700 °C.

The untreated sample (initial sample, Figure 3a) does not have any oxides or superficial layers, but Figure 3d,e (corresponding to 400 °C and 500 °C temperature treatment) shows a superficial layer that could be identified as ferrous oxide, corresponding also to the dark-brown color detected in the visual analysis of these samples. Nevertheless, this study does not provide information on the bluish color present on the sample treated at 300 °C, neither can it guarantee the supposed pre-identified oxide layer on the other samples. To complement this study, an SEM microstructure analysis is performed.

**Figure 4:**
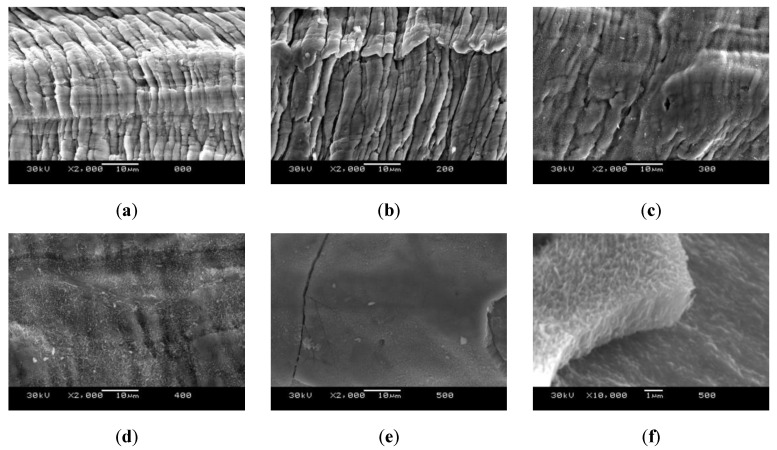
Scanning electro-microscopy (SEM) analysis of the wool samples after thermal treatment under an inert atmosphere. (**a**) Initial sample, (**b**) sample treated at 200 °C, (**c**) sample treated at 300 °C, (**d**) sample treated at 400 °C, (**e**) sample treated at 500 °C, and (**f**) detail of the superficial layer of the sample treated at 500 °C.

The results of the SEM microstructure analysis is shown in Figure 4. The fluted surface seen in Figure 4a,b is the one expected for the production process due to the deformation of the fiber when it is extracted from the matrix. This structure is expected to be found in all the samples since the treatment temperature was kept below the recrystallization temperature of steel, as explained above. These samples also show an absence of any kind of external layer or compound.

The change in the surface of the sample treated at 300 °C (Figure 4c), with the formation of small filaments covering the fluted surface, is characteristic of the incipient formation of metallic oxides. In this sample, the covered surface is small, and the fluted surface can still be observed. After the treatment at 400 °C (Figure 4d), the changes cover the whole surface, yet allowing to suspect the presence of the fluted surface behind. Finally, in the sample treated at 500 °C (Figure 4e), a dense layer with an average thickness of 2 µm can be appreciated over the surface, completely covering the fluted surface. Figure 4f shows that this layer is not adhered to the surface, indicating a morphology similar to that of oxides.

**Figure 5:**
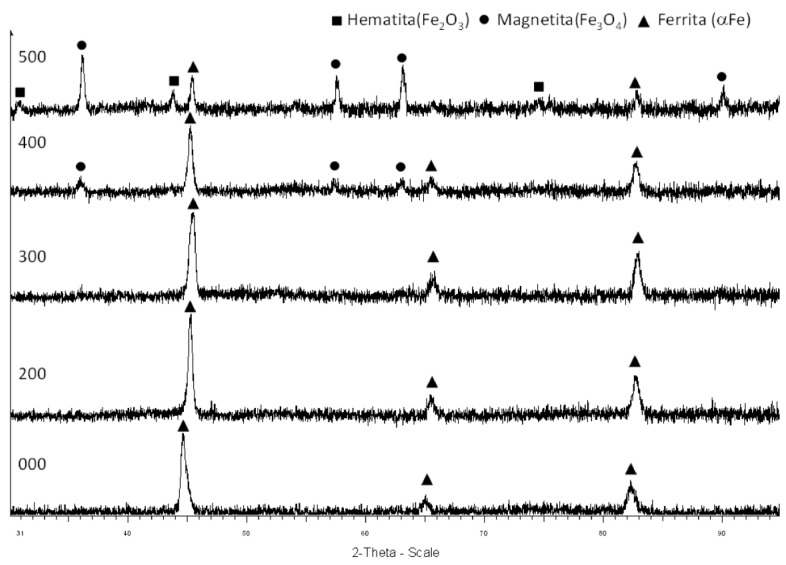
Crystalline phases of the wool samples after thermal treatment under an inert atmosphere. From bottom to top: Initial sample, sample treated at 200 °C, sample treated at 300 °C, sample treated at 400 °C, and sample treated at 500 °C.

The DRX analysis allows the determination of the crystalline structure of the studied samples (Figure 5). The untreated sample has a crystalline structure characteristic of ferrite. The crystalline structure of the samples treated at 200 °C and 300 °C is similar, as expected according to the SEM images (Figure 4b,c), showing that the filaments seen in the micrographs can be identified as ferrous oxides. At 400 °C, the DRX shows peaks corresponding to magnetite (Fe_3_O_4_), which corresponds to the oxide layer seen in Figure 4d. Finally, the DRX of the sample treated at 500 °C shows magnetite and hematite (Fe_2_O_3_), which corresponds to the color of the sample seen in Figure 2.

## 3. Materials and Methods

### 3.1. Material

The metal wool used for the experiments was obtained by cutting a metal cable with blades, as depicted in [17]. The metal was stainless steel AISI 424 from Barlesa© (Oggiono, Italy) with a density of 199 kg/m^3^, a pure steel density of 7800 kg/m^3^, and a porosity of 0.97 ppu. The fibers had a diameter between 10 and 300 µm and thermal conductivity of 40 W/m·K.

To achieve maximum neutrality vs. the PCM to be used, in these experiments, the metal wool was mixed with an epoxy resin.

### 3.2. Production of the Metal Wool Composite

For the experiments, two types of samples were prepared, the first one using casting methods and the second one using vacuum infiltration. The original fibers present a tangle configuration, with the surface layers presenting different orientations. To reduce this randomness of fibers and increase their directionality, pretreatment is carried out in which the surface layers with less directionality are extracted, leaving the central oriented part of the metal wool fibers.

**Figure 6:**
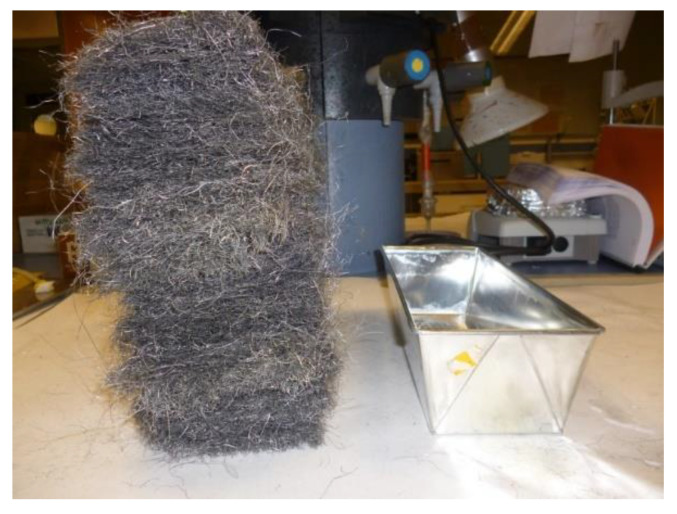
Metal wool and mold used in the casting laboratory trials.

**Figure 7:**
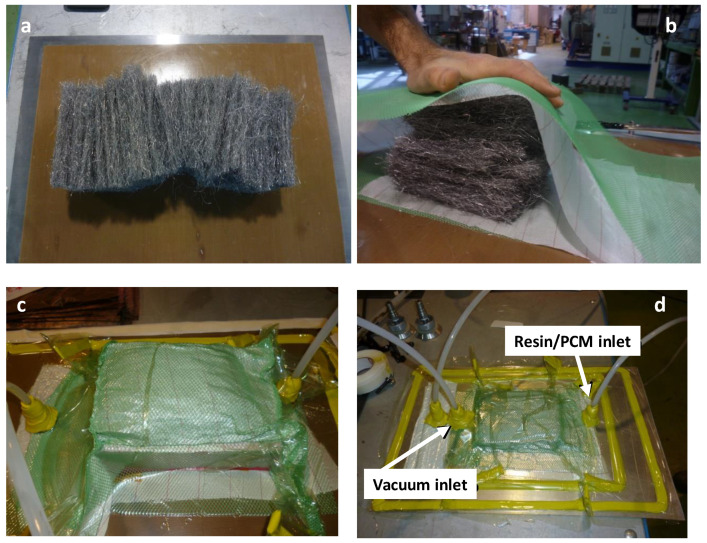
Production of metal wool-resin compound. (**a**) Metal fibers, (**b**) insertion in plastic bags, (**c**) vacuum production, and (**d**) resin infiltration.

In the first sample, the metal wool was inserted in a metal container and compacted following a casting process. In order to obtain a proportion of 10% fiber:90% Epoxi Resoltech 1050 resin in volume, the amount of fibers was calculated using 73 g of metal wool in a mold of 275 × 85 × 75 mm (Figure 6). To proceed with the casting, the liquefied PCM was inserted at 140 °C in the container with the fibers previously pre-heated to 170 °C. This temperature was kept for 10 min to allow the correct curing of the resin and then allowed to cool down to ambient temperature. For the second sample, the composite was produced using vacuum infiltration. To do so, the metal wool was inserted in a plastic bag, then a vacuum was produced, and finally, the resin was infiltrated (Figure 7) and heated up for curing. This process is similar to those commercially used to produce laminated glass panels [22].

### 3.3. Effective Thermal Conductivity Evaluation

**Figure 8:**
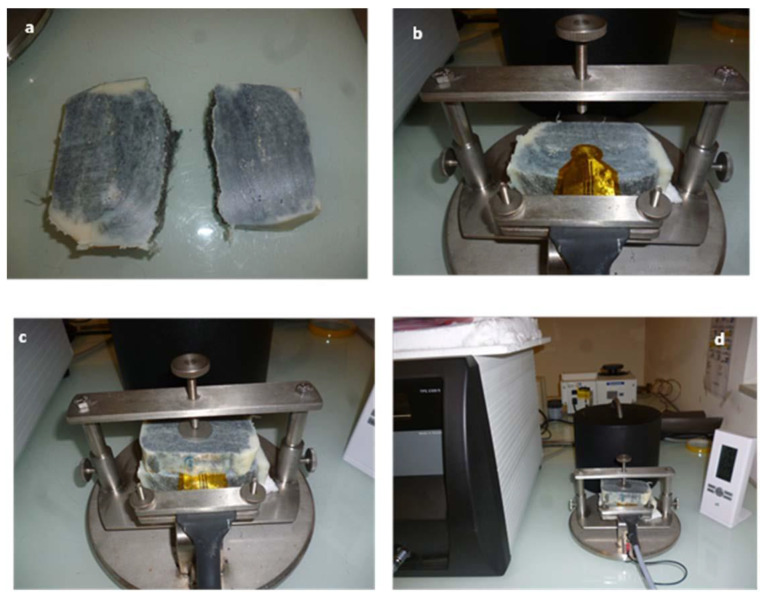
Hot disc method. (**a**) Samples to be tested, (**b**) Kapton sensor to the used, (**c**) Kapton sensor between to test samples, and (**d**) full equipment.

The thermal conductivity of the samples is measured using a Hot Disk 2500S equipment with Kapton sensors (Donostia-San Sebastián, Spain) for the determination of the thermal conductivity using the transient plane source hot-disk technique [23]. The basic principle of this method relies on a plane element that acts both as a temperature sensor and heat source [24,25]. This element consists of an electrically conducting pattern of thin nickel foil (10 μm) spiral-shaped, embedded in an insulating layer usually made of Kapton (70 μm thick). The HD probe is located between two samples with both sensor faces in contact with the two samples’ surfaces of similar characteristics. This equipment can measure materials with different geometries and properties at ambient temperature (Figure 8). The thermal conductivity was measured in accordance with ISO 22007-:2017 and ISO 22007-2:2015 by sandwiching an electrically insulated flat disk-shaped nickel sensor in between two identical samples of wool-based composite materials. The sensor behaves as a heat source and a temperature monitor simultaneously. The surfaces of the prepared epoxy matrix composite materials are flattened and cleaned to reduce the contact resistance between the sensor-sample surfaces. The thermal conductivity of the conducting thin-films of the prepared composite materials in the direction parallel to the carbon layers is measured using a comparative technique [26]. Thermal conduction in the prepared thermal interface materials and composite materials is found to be anisotropic since the difference between the thermal conductivities measured along three mutually perpendicular axes is significant. The selection of three mutually perpendicular axes is made based on the pressure loading direction during the preparation process of thermal interface materials and composite materials. There is no significant difference in thermal conductivity between the two directions that are perpendicular to the pressure loading direction.

This method requires the use of two samples with the same dimensions, with the sensor located between them. To accomplish this, two 20 mm thick samples are cut and the contact surfaces polished. For the generation of the samples, the direction of the fibers is considered, differentiating between axial and radial configuration. The following images (Figure 8) show the methodology used for the axial thermal conductivity analysis, with the samples cut perpendicularly to the fibers.

**Figure 9:**
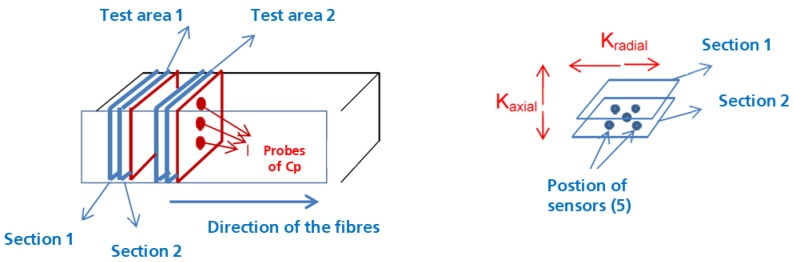
Effective thermal conductivity measurement.

The material has been characterized considering that it has both isotropic and anisotropic behavior [27]. The equipment used for the experimentation can report information on the thermal conductivity, the thermal diffusivity and the calorific value using the measurement method of isotropic materials, while for anisotropic materials, the calorific value is an external piece of data and the equipment provides only the thermal diffusivity and conductivity. The anisotropy of the composite is taken into consideration by inserting the measuring probes in two sections in the direction of the heat transfers, as shown in Figure 9. This method requires using the calorific value (Cp) to later calculate the thermal conductivity [28].

As it can be seen in Figure 9, for each section of the samples, the Cp is measured in probes of 12.5 mm of diameter and 3 mm thickness. For these probes, the density is also measured. These values are displayed in Table 3 and Table 4.

The technical datasheet of the resin was used to establish a value of 1.17 g/cm^3^ for its density, while the thermal conductivity is not normally displayed. Nevertheless, this parameter can be found in the bibliography, with values around 0.2 W/m·K.

With the data available for the specific heat value, an analysis of the material was carried out considering its anisotropic nature. For every specific heat value, different measurements were made and the axial thermal conductivity (in the alignment direction of the metallic fibers) and radial (in the plane perpendicular to the metallic fibers) were evaluated (Table 1 and Table 2).

### 3.4. Behavior of the Metal Wool under an Inert Atmosphere

The final configuration of the composite formed by the chosen PCM and the metal fiber will be selected depending on the target process temperature at which the storage works. For every composite, studies must be carried out to guarantee the compatibility of the materials used. However, it is important to validate that the wool is not going to degrade by thermal cycling within the temperature range in which this solution can have industrial applications. For industrial storage solutions, the range between 200 °C and 500 °C is selected [18].

A first characterization of the wool samples without composite was performed, analyzing its behavior at high temperatures under an inert atmosphere. The procedure for this analysis is explained below.

**Figure 10:**
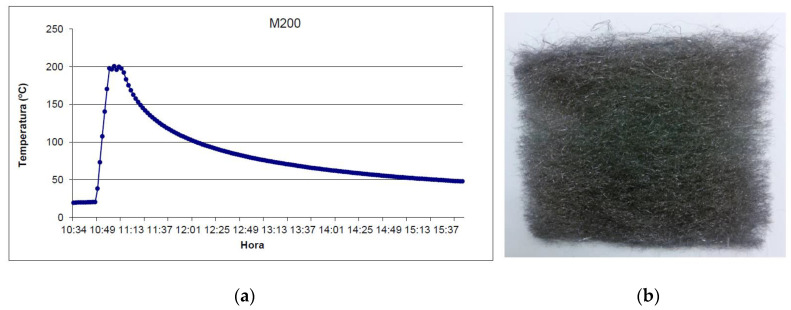
(**a**) Temperature-time process for the test at 200 °C and (**b**) sample after the testing.

Wool samples with 10 × 10 cm dimension (Figure 10b) were heated up in a CCI electric furnace at 200 °C, 300 °C, 400 °C, and 500 °C under an inert nitrogen atmosphere. The samples were first introduced in the furnace, then the furnace was filled with nitrogen, and finally, the temperature of the furnace was raised to the temperature objective. The temperature was then maintained for 10 min, after which the furnace was left to cool down to room temperature, as can be seen in Figure 10a. At this point, the experiment was considered to have finished. This procedure was repeated for all samples at the desired temperature (200 °C, 300 °C, 400 °C, and 500 °C).

The treated samples were analyzed firstly with an optical microscope under low magnification and secondly with a morphological analysis using scanning electron microscopy (SEM). After those analyses, the samples were chemically characterized by XRD. The equipment used in this study was an SEM-JEOL 5910-LV microscope (Seville, Spain) and a Brucker D8-Advance XRay-Diffractometer (Seville, Spain).

## 4. Conclusions

This paper shows that it is possible to use metal wool as a low-cost thermal conductivity enhancement technique for latent TES in solar process heat applications. To achieve an effective thermal conductivity, the PCM should be impregnated in the metal wool under vacuum conditions. This study used a glass panel commercial process, ensuring its deployment into the industry. The storage composite obtained has an anisotropic effective thermal conductivity similar to graphite composites. The measured effective thermal conductivity of the storage composite is 4.34 W/m·K, similar to the values considered as adequate by the industry.

Additionally, tests were performed to validate the thermal degradation of metal wool for high thermal cycling temperatures. The tests were carried out at 200 °C to 500 °C in an inert atmosphere. The results show that the metal wool does not present any chemical or physical degradation, once again demonstrating that this thermal conductivity enhancement technique is adequate for the considered application.

For the commercial application of this configuration, the selection of the PCM will be performed in accordance with the specific process and the storage temperature, and additional corrosion studies must be performed with the selected composite.

## Figures and Tables

**Table 1:** Measured axial effective thermal conductivity.

Sample	Sample 1	Sample 2	Sample 3	Sample 4	Sample 5	Average
Section 1	4.08	4.62	4.90	4.84	4.89	4.66
Section 2	3.95	4.56	3.90	4.50	4.70	4.31

**Table 2:** Measured radial effective thermal conductivity.

Sample	Sample 1	Sample 2	Sample 3	Sample 4	Sample 5	Average
Section 1	0.80	0.80	0.73	0.75	0.72	0.76
Section 2	0.77	0.71	0.77	0.82	0.89	0.77

**Table 3:** Measured density.

Sample	Density 1 (g/cm^3^)	Density 2 (g/cm^3^)	Density 3 (g/cm^3^)	Average (g/cm^3^)
Section 1	1.77	1.83	1.76	1.79
Section 2	1.81	1.77	1.69	1.76

**Table 4:** Measured calorific value (Cp).

Sample	Cp 1 (MJ/m^3^K)	Cp 2 (MJ/m^3^K)	Cp 3 (MJ/m^3^K)	Average (MJ/m^3^K)
Section 1	1.75	1.82	1.60	1.72
Section 2	1.55	1.64	1.6	1.6

## Data Availability

The data presented in this study are available on request from the corresponding authors.

## Sample Availability

Samples of the study are available on request from the corresponding authors.

## References

[1] Crespo A., Barreneche C., Ibarra M., Platzer W. (2019). Latent thermal energy storage for solar process heat applications at medium-high temperatures—A review. Sol. Energy.

[2] Yang G., Yim Y.-J., Lee J.W., Heo Y.-J., Park S.-J. (2019). Carbon-Filled Organic Phase-Change Materials for Thermal Energy Storage: A Review. Molecules.

[3] AboKersh M.H., Osman M., El-Baz O., El-Morsi M., Sharaf O. (2018). Review of the phase change material (PCM) usage for solar domestic water heating systems (SDWHS). Int. J. Energy Res..

[4] Kee S.Y., Munusamy Y., Ong K.S. (2018). Review of solar water heaters incorporating solid-liquid organic phase change materials as thermal storage. Appl. Therm. Eng..

[5] Pandey A., Hossain M., Tyagi V., Rahim N.A., Selvaraj J.A., Sari A. (2018). Novel approaches and recent developments on potential applications of phase change materials in solar energy. Renew. Sustain. Energy Rev..

[6] Prieto C., Cabeza L.F. (2019). Thermal energy storage (TES) with phase change materials (PCM) in solar power plants (CSP). Concept and plant performance. Appl. Energy.

[7] Sharan P., Turchi C., Kurup P. (2019). Optimal design of phase change material storage for steam production using annual simulation. Sol. Energy.

[8] Abujas C.R., Jové A., Prieto C., Gallas M., Cabeza L.F. (2016). Performance comparison of a group of thermal conductivity enhancement methodology in phase change material for thermal storage application. Renew. Energy.

[9] Gasia J., Miró L., Cabeza L.F. (2016). Materials and system requirements of high temperature thermal energy storage systems: A review. Part 2: Thermal conductivity enhancement techniques. Renew. Sustain. Energy Rev..

[10] Steinmann W.-D., Laing D., Tamme R. (2010). Latent Heat Storage Systems for Solar Thermal Power Plants and Process Heat Applications. J. Sol. Energy Eng..

[11] Ikutegbe C.A., Farid M.M. (2020). Application of phase change material foam composites in the built environment: A critical review. Renew. Sustain. Energy Rev..

[12] Cabeza L.F., Ibáñez M., Solé C., Roca J., Nogués M. (2006). Experimentation with a water tank including a PCM module. Sol. Energy Mater. Sol. Cells.

[13] Mahdi J.M., Nsofor E.C. (2018). Multiple-segment metal foam application in the shell-and-tube PCM thermal energy storage system. J. Energy Storage.

[14] Gasia J., Maldonado J.M., Galati F., De Simone M., Cabeza L.F. (2019). Experimental evaluation of the use of fins and metal wool as heat transfer enhancement techniques in a latent heat thermal energy storage system. Energy Convers. Manag..

[15] Yousef M.S., Hassan H. (2019). An experimental work on the performance of single slope solar still incorporated with latent heat storage system in hot climate conditions. J. Clean. Prod..

[16] Yousef M.S., Hassan H. (2019). Energetic and exergetic performance assessment of the inclusion of phase change materials (PCM) in a solar distillation system. Energy Convers. Manag..

[17] Prieto C., Rubio C., Cabeza L.F. (2021). New phase change material storage concept including metal wool as heat transfer enhancement method for solar heat use in industry. J. Energy Storage.

[18] Yang J., Yang W., Chen W., Tao X. (2020). An elegant coupling: Freeze-casting and versatile polymer composites. Prog. Polym. Sci..

[19] Liu R., Zhang F., Su W., Zhao H., Wang C.-A. (2015). Impregnation of porous mullite with Na_2_SO_4_ phase change material for thermal energy storage. Sol. Energy Mater. Sol. Cells.

[20] Maldonado J.M., Fernández Á.G., Cabeza L.F. (2019). Corrosion assessment of myo-inositol sugar alcohol as a phase change material in storage systems connected to Fresnel solar plants. Molecules.

[21] Chen J., Gao X. (2019). Directional dependence of electrical and thermal properties in graphene-nanoplatelet-based composite materials. Results Phys..

[22] Martín M., Centelles X., Solé A., Barreneche C., Fernández A.I., Cabeza L.F. (2020). Polymeric interlayer materials for laminated glass: A review. Constr. Build. Mater..

[23] Zhao W., Yang Y., Bao Z., Yan D., Zhu Z. (2020). Methods for measuring the effective thermal conductivity of metal hydride beds: A review. Int. J. Hydrogen Energy.

[24] Gustafsson S.E. (1991). Transient plane source techniques for thermal conductivity and thermal diffusivity measurements of solid materials. Rev. Sci. Instrum..

[25] Gustavsson M., Karawacki E., Gustafsson S.E. (1994). Thermal conductivity, thermal diffusivity, and specific heat of thin samples from transient measurements with hot disk sensors. Rev. Sci. Instrum..

[26] Biercuk M.J., Llaguno M.C., Radosavljevic M., Hyun J.K., Johnson A.T., Fischer J.E. (2002). Carbon nanotube composites for thermal management. Appl. Phys. Lett..

[27] Madrid F. (2005). Thermal Conductivity and Specific Heat Measurements for Power Electronics Packaging Materials. Effective Thermal Conductivity Steady State and Transient Thermal Parameter Identification Methods.

[28] Han D., Yue K., Cheng L., Yang X., Zhang X. (2000). Measurement of the Thermophysical Properties of Anisotropic Insulation Materials with Consideration of the Effect of Thermal Contact Resistance. Materials.

